# The Impacts of COVID-19 on China’s Economy and Energy in the Context of Trade Protectionism

**DOI:** 10.3390/ijerph182312768

**Published:** 2021-12-03

**Authors:** Feng Wang, Min Wu

**Affiliations:** School of Economics and Finance, Xi’an Jiaotong University, Xi’an 710061, China; wangfeng123@xjtu.edu.cn

**Keywords:** COVID-19, trade protectionism, economy, energy, input-output model

## Abstract

In the current context of rising trade protectionism, deeply understanding the impacts of COVID-19 on economy and energy has important practical significance for China to cope with external shocks in an uncertain environment and enhance economic resilience. By constructing an integrated economic and energy input-output model including the COVID-19 shock, this paper assesses the impacts of COVID-19 on China’s macro-economy and energy consumption in the context of trade protectionism. The results are shown as follows. First, in the context of protectionism, the outbreak of COVID-19 in China would cause a 2.2–3.09% drop in China’s GDP and a 1.56–2.48% drop in energy consumption, while adverse spillovers from global spread of COVID-19 would reduce its GDP by 2.27–3.28% and energy consumption by 2.48–3.49%. Second, the negative impacts of domestic outbreak on China’s construction, non-metallic mineral products, and services would be on average 1.29% higher than those on other industries, while the impacts of global spread of COVID-19 on export-oriented industries such as textiles and wearing apparel would be on average 1.23% higher than other industries. Third, the effects of two wave of the pandemic on China’s fossil energy consumption would be on average 1.44% and 0.93% higher than non-fossil energy consumption, respectively.

## 1. Introduction

Since the outbreak of the 2008 financial crisis, trade protectionism has gradually risen in the international market [1,2]. According to Global Trade Alert (GTA), China has experienced the highest number of protectionist measures. The coronavirus disease (COVID-19) outbreak was discovered in Wuhan, China, in December 2019 and then spread rapidly to multiple countries around the world in early 2020, which was characterized as a pandemic by the World Health Organization (WHO). The COVID-19 pandemic not only has a direct impact on China’s economy, but also entails disruptions of global value chains and recessions in major economies, thus exposing China to adverse global spillovers. Meanwhile, the panic caused by the pandemic may further exacerbate global trade protectionism [3,4]. This shows that the COVID-19 pandemic poses a huge challenge to China’s economy in the context of trade protectionism. In addition, some scholars found that the pandemic may also affect energy consumption, which may be due to a bidirectional causality between energy consumption and economic growth [5,6]. For example, Smith et al. [7] argued that the pandemic would cause a decline in energy consumption in major carbon-emitting countries. Norouzi et al. [8] found that the pandemic has delivered a shock to electricity and oil demand in China. Similarly, Wang and Su [9] suggested that the reductions in economic activity and the restrictions on transport caused by COVID-19 has significantly decreased China’s energy consumption, especially coal consumption. The above evidence indicates that the COVID-19 pandemic will not only bring substantial challenges to China’s economy but also affect its energy consumption.

Much of the current literature on the pandemic analyzes its social and economic impacts, such as its impact on output [10], industry volatility [11] and interest rates [12]. Other literature focuses on the effects of the pandemic on energy and environment, such as its impact on energy consumption [13] and air pollution [14,15]. However, most researchers focusing on the economic impacts of COVID-19 ignored the effects of the pandemic on the flow of energy products [10,11,12], while those examining the impacts of the pandemic on energy consumption rarely considered the relationship between energy and economy [13,14,15]. In addition, no studies have been found in the searchable literature that analyze the impact of the pandemic on China in the current context of rising trade protectionism. Currently, China is experiencing the shock of COVID-19 in the context of rising trade protectionism. Combined with the international context that China is facing, deeply understanding the effects of the pandemic on China’s economy and energy in this context is of great significance for China to respond to external shocks in an uncertain environment, enhance economic resilience, safeguard national security, and promote high-quality development. Therefore, this paper will evaluate the impacts of the COVID-19 shock on China’s economy and energy in the context of trade protectionism. Specifically, this paper will first construct an integrated economic and energy input-output (IEEIO) model including the COVID-19 shock based on the characteristics of such shock. This model can capture the changes in the global economic supply chain and energy conversion chain under the pandemic shock. Then, according to the development of the pandemic, we will set scenarios to simulate the shock of the COVID-19 outbreak in China and the shock of the COVID-19 global spread. Finally, based on the IEEIO model, including the COVID-19 shock and related scenarios, this paper will simulate and evaluate the impacts of the outbreak and spread of the pandemic on China’s macro-economy, industry outputs, and energy consumption in the context of trade protectionism.

This paper makes three contributions to the literature. First, this paper is the first to assess the impacts of the COVID-19 shock on China’s economic development, industry outputs, and energy flows from the perspective of economic–energy interactions. Although some researchers have evaluated the effects of the COVID-19 pandemic on the economy or on energy [10,11,12,13,14,15], most of them focus on a single dimension, and there is a lack of studies that comprehensively examine the impacts of the pandemic on the macro-economic level and on energy flows from the perspective of economic–energy interactions. Second, fully considering the nature and characteristics of the shock of COVID-19, we introduce the pandemic’s impact on supply-side and demand-side in different forms into the IEEIO model, thus constructing the IEEIO model including the COVID-19 shock. This model can capture the changes from the shock of COVID-19 in global supply chains and energy conversion chains. Third, given the characteristics of the outbreak and spread of the pandemic and its uncertainty, this paper innovatively sets up 11 scenarios to simulate and extrapolate the impacts of the pandemic on China’s economy and energy in the context of trade protectionism.

The structure of this paper is as follows: Section 2 reviews the relevant literature; Section 3 constructs an IEEIO model including the COVID-19 shock; Section 4 introduces the design of scenarios and data sources; Section 5 presents the results and discussion; and the last section provides the conclusions and policy implications.

## 2. Literature Review

From the existing literature, there are three main types of literature germane to this paper: the first is the literature that assesses the effects of COVID-19; the second is the literature that examines the economic and energy impacts of COVID-19 on China; the third is the literature analyzing the impacts of COVID-19 on the world and other countries.

A variety of methods have been used in the literature to assess the impacts of COVID-19, methods which could be broadly classified into three categories. One of the common methods is the computable general equilibrium (CGE) model. This model can be used to evaluate the effects of the pandemic from a macro and comprehensive perspective [16]. Based on global hybrid dynamic stochastic general equilibrium (DSGE)–computable general equilibrium (CGE) general equilibrium model, McKibbin and Fernando [17] and Jawad et al. [18] predicted the possible progress of COVID-19 in seven scenarios and assessed the macroeconomic impacts of the pandemic under each scenario. Madai Boukar et al. [19] used the CGE model to evaluate the effects of COVID-19 on employment in Cameroon’s different sectors. The CGE model can identify all economic activities in a consistent way, in theory, reflecting the interdependence of economic sectors [20]. However, the modeling of the CGE model is complex, and the sensitivity of CGE outputs to shocks, model types, and closure rules may hinder the applicability of this paper to impact assessments of structural changes caused by shocks [21]. The input-output model is another common method for evaluating the impacts of the pandemic on economy and energy. Based on the input-output model, Sayan and Alkan [22] and Bonet-Morón et al. [23] assessed the economic costs of the pandemic control measures, while Huang and Tian [24] analyzed the impacts of the pandemic on inequality in carbon emissions. The input-output model has been simplified to the easily constructed inter-industry-based tables [25], which is suitable for capturing the impacts of sudden shocks on the economy [20]. However, this model has the limitations that the technical coefficient is assumed to be constant, the production function is assumed to be linear, and it is only applicable to static analysis. The third type of common methods for assessing the impact of the pandemic is the econometric model. Using econometric models, Aruga et al. [26] examined the impacts of COVID-19 on energy consumption in India, Shaikh [27] revealed the effects of the COVID-19 on energy markets, and Iqbal et al. [28] assessed the impacts of the pandemic on energy consumption and carbon emissions. The econometric model can reflect the historical trend of the economy and the schedule of economic impact, but it is constrained by the nature of past economic relations and cannot predict possible changes in economic events or activities. In conclusion, since the COVID-19 shock is a sudden short-term shock, the input-output model that is relatively simple and more suitable for assessing shock bursts is more appropriate for this study.

From the emergence of COVID-19 in China, many researchers have begun to examine the domestic impact of the pandemic. Relevant studies mainly focus on the social and economic impacts of the pandemic on China, as well as the energy and environmental impacts. In terms of the social and economic impacts, Zhou et al. [29] and Hu et al. [10] evaluated the macroeconomic effects of COVID-19 on China using the CGE model, and found that the pandemic had heterogeneous impacts on industrial outputs, and the impact on the secondary industry was significantly greater than that on the tertiary industry. Taking a different approach, Duan et al. [30] adopted a quarterly CGE model to assess the economic impacts of COVID-19 on China at the national and industrial levels, and suggested that the service sector was most affected by the pandemic; Tan et al. [31] also found that firms and activities related to the service sector were most affected. Regarding the impacts of COVID-19 on energy and environment in China, related studies found that the pandemic is reducing energy consumption and pollutant emissions [9,32]. Specifically, the electricity demand [8,33] and oil demand [8] in China were found to be severely affected by the pandemic. However, Wang and Su [9] suggested that energy consumption and greenhouse gas emissions might exceed the prepandemic levels when China resumes large-scale industrial production. Furthermore, some scholars also focused on the changes in China’s economy, energy and environment during the pandemic. For example, Xu et al. [34] examined the causal relationship between economic development and environmental quality during this public health crisis. Their results indicated that economic activities mainly caused environmental pollution and energy use through the COVID-19 shock in China. Jia et al. [35] also suggested that the decline of global carbon emissions caused by the pandemic was only due to economic recession.

As COVID-19 rapidly spread internationally, many scholars have also studied the impacts of the pandemic on the world, as a whole, and in other countries individually. Research on the global level focuses on the pandemic’s impacts on the macroeconomic and microeconomic levels [17,36] as well as on the social economy [37], environment [38], and energy [39,40]. Related studies found that the pandemic hit the global economy significantly [17], caused huge losses of economic well-being and social capital [37], and also severely impacted energy and environmental sectors [39,40]. Some scholars have also examined the economic impacts of COVID-19 on a range of countries around the world. Salisu et al. [41] and Chudik et al. [42] found that the pandemic had negative effects on the economies of many countries to varying degrees, with more profound and lasting effects on developed economies than emerging economies. In addition, some scholars have conducted studies on some countries where the pandemic was more serious, and assessed the economic shocks of the pandemic on the United States [43,44], Britain [45], India [46,47], Australia [48], Italy [45] and Canada [49] as well as its impacts on energy demand and energy consumption in the United States [50], India [26] and Canada [51].

In summary, the methods for evaluating the pandemic’s impacts used by most of the literature fail to describe in detail the changes in energy conversion chain under the shock, and fail to incorporate the interaction between energy and economy. Moreover, studies on the effects of COVID-19 on China usually examine only its economic or energy impacts. There remains a paucity of research on assessing comprehensively the impacts of the pandemic on economic growth, industry development, and energy flows. Therefore, we will construct an IEEIO model including the COVID-19 shock, and evaluate the impacts of the shock on China’s economic growth and energy flows in the context of trade protectionism.

## 3. Methods

The IEEIO model was constructed by our research group [52]; it can be used to assess the impacts of external shocks on China’s economy and energy. Due to space limitations, this paper will briefly introduce the basic IEEIO model and explain how to construct the IEEIO model including the COVID-19 shock.

### 3.1. Basic IEEIO Model

The IEEIO model is constructed by integrating the global multi-regional input-output (GMRIO) model and the global energy multi-regional supply and use (GEMRSU) model. The introduction of the GMRIO model and the GEMRSU model is shown in Appendix A. Then, we will introduce the IEEIO model.

The link between the GMRIO model and the GEMRSU model is established by the energy products use intensity matrix *T* of non-energy industries. By collation, the total outputs of energy products *E* can be expressed as:

(1) $$E=L_{E}TL_{n}Y\cdot e+L_{E}H_{E}\cdot e$$

where *L_E_* is the energy product total requirements matrix of energy industries. *T* refers to the energy products use intensity matrix of non-energy industries. *L_n_* denotes the submatrix of Leontief inverse matrix *L*, *L* = (*I* − *A*)^−1^, representing the total requirements matrix for non-energy products in each industry. *Y* is the final demand matrix. *H_E_* denotes the final demand matrix of energy products for households and *e* refers to the summation vector consisting entirely of ones.

### 3.2. The IEEIO Model including the COVID-19 Shock

This paper constructs an IEEIO model including the COVID-19 shock to assess the pandemic’s impacts on China’s economy and energy in the context of trade protectionism. First, this paper incorporates the context of protectionism into models by changing the trade relations among regions. This is done by changing the data associated with trade, as described in more detail in Wang and Wu [52]. Then, as the pandemic weighs on both demand and supply, we incorporate the COVID-19 shock into economic model by changing the final demand structure and adding supply constraints in the optimization problem. The pandemic shock will be further transmitted from economic system to energy system through the IEEIO model.

The procedure for introducing the COVID-19 shock into models is as follows.

(1) Add supply constraints in the optimization problem to introduce the supply shock arising from COVID-19, change final demand structure *s* to introduce the demand shock, and solve the optimization problem including the impacts on obtaining the final demand *Y* (in monetary units) and the value added *V* (in monetary units) in the optimal solution;
(2) Estimate the total outputs of energy products *E* (in energy units) using the IEEIO model including the COVID-19 shock;
(3) Compare the value added *V* (in monetary units) and the total outputs of energy products *E* (in energy units) estimated in this scenario with those in the baseline scenarios including trade protectionism to obtain the pandemic’s economic and energy impacts in the context of trade protectionism.

It is worth noting that since the IEEIO model is constructed based on the input-output model, it also suffers from that model’s same limitations, which mainly include three aspects: first, the technical coefficient is assumed to be a constant; second, the production function is assumed to be linear; and third, this model is only applicable to static analysis. Next, we will describe the effects of these limitations on interpreting results. First, the assumption of the constant technical coefficient is relatively reasonable for the study in this paper. Since this paper aims to analyze the short-term effects of COVID-19 on China’s economy and energy, technology could be assumed to be constant in the short term. Second, the assumption of the linear model does have a certain impact on the research of this paper. In fact, the pandemic’s impacts on China’s economy and energy might be nonlinear. However, it is difficult to capture these nonlinear impacts and to characterize them accurately. Therefore, to simplify the analysis, this paper simulates the shock of the pandemic based on the linear assumption, which is relatively reasonable and could provide a good benchmark for the evaluation of this shock. Finally, although the input-output model is only suitable for static analysis, it is feasible to use this model to evaluate the effects of the pandemic on China’s economy and energy because the pandemic shock simulated in this paper is a sudden short-term shock.

## 4. Scenarios and Data

Based on the IEEIO model including the COVID-19 shock, this paper defines various scenarios to simulate and assess the pandemic’s economic and energy impacts on China in the context of trade protectionism. In addition, the data for the COVID-19 pandemic is introduced in this section, while the data for the GMRIO table and GEMRSU table is shown in Appendix B. According to the number published by GTA of discriminatory trade restrictions implemented by countries against China, this paper divides countries covered by the World Input-Output Table (WIOT) into three trade regions: China; countries with many discriminatory trade restrictions against China (simply CTR hereafter, including the United States, India, Germany, Brazil, and Canada); and rest of the World (simply ROW hereafter).

### 4.1. Detailed Information about Incorporating the COVID-19 Pandemic into Models

The COVID-19 pandemic could be roughly divided into two waves based on its emergence and spread. The initial wave of the pandemic refers to the outbreak of COVID-19 in China (mainly in the first quarter of 2020), and the second wave is the global spread of COVID-19.

(1) The initial wave of the pandemic

The initial wave of the pandemic delivered a shock to China’s economy. This paper introduces this shock into the economic model of supply and demand. On the supply side, the pandemic control measures reduce labor supply and disrupt transportation, thus lowering productivity. Therefore, we add supply constraints in the optimization problem shown in Appendix A.1 to introduce the supply shock arising from the domestic outbreak:

(2) $$X_{i}^{CHN}\leq (1-\alpha_{i}^{CHN})X_{i}^{CHN,0}$$

where *X_i_^CHN^* is the element of total outputs vector *X*, representing the output of industry *i* in China. *X_i_^CHN^*^,0^ refers to the baseline value of the output of industry *i* in China. *α_i_^CHN^* represents the productivity loss rate of industry *i* in China under domestic outbreak.

On the demand side, the pandemic not only decreases consumption of wholesale and retail, accommodation and food service, travel and other services, but also negatively affects investment. Since the pandemic shock on consumption and investment in China will cause changes in final demand structure, we introduce the demand shock arising from domestic outbreak by changing final demand structure in the optimization problem. The specific steps are as follows. First, estimate the decline rate of China’s final demand for products of each trade region. Second, estimate final demand of each trade region. Finally, recalculate the final demand structure *s* under domestic outbreak.

(2) The second wave of the pandemic

The pandemic continues to spread across the world although the pandemic in China has been brought under control. CTR and ROW economies have been hit by the second wave of the pandemic; therefore, this paper introduces the impacts of the global spread of COVID-19 on these economies into the economic model through supply- and demand-side. On the supply side, the spread of the pandemic would have a direct negative impact on production activities in these two regions. We incorporate the supply shock arising from global spread of the pandemic on these regions by adding supply constraints in the optimization problem:

(3) $$X_{j}^{CTR}\leq (1-\alpha_{j}^{CTR})X_{j}^{CTR,0},\;X_{k}^{ROW}\leq (1-\alpha_{k}^{ROW})X_{k}^{ROW,0}$$

where *X_j_^CTR^* and *X_k_^ROW^* refer to the elements of total outputs vector *X*, which represent the outputs of industry *j* in CTR and industry *k* in ROW, respectively. *X_j_^CTR^*^,0^ and *X_k_^ROW^*^,0^ are the baseline values of *X_j_^CTR^* and *X_k_^ROW^*. *α_j_^CTR^* and *α_k_^ROW^* represent the productivity loss rates of industry *j* in CTR and industry *k* in ROW, respectively.

On the demand side, global spread of the pandemic delivers a negative shock to consumption and investment in CTR and ROW. This is reflected in the optimization problem as the changes in final demand structure. Thus, we reestimate the final demand structure to introduce the demand shock arising from the spread of the pandemic on CTR and ROW into the economic model. The estimation steps of final demand structure under the spread of the outbreak are basically consistent with that under domestic outbreak.

### 4.2. Design of Scenarios

**Figure 1:**
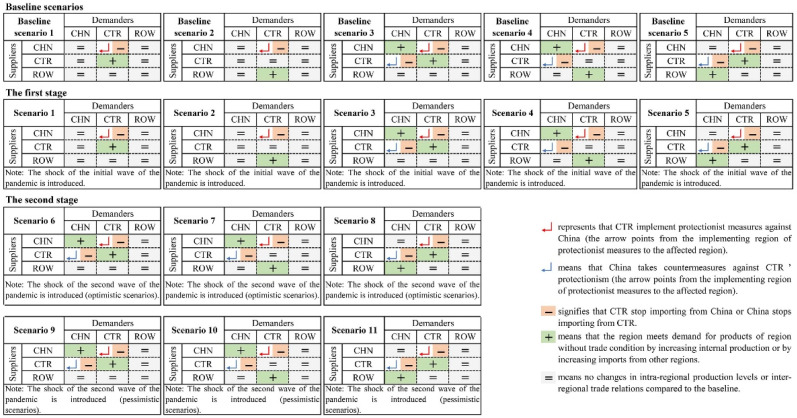
Design of baseline scenarios and scenarios for COVID-19.

Since the purpose of this paper is to simulate and assess the impact of COVID-19 on China’s economy and energy in the context of trade protectionism, we set the baseline scenarios to include the context of trade protectionism. In order to cope with the uncertainty of trade policies across regions, this paper sets up five baseline scenarios (baseline scenarios 1–5 in Figure 1) based on the extreme trade relations among regions that may be caused by trade protectionism.

Next, to facilitate the scenario analysis, we divide the shock of COVID-19 into two stages according to the development of the pandemic: the first stage is assumed to be the stage with the outbreak of COVID-19 in China, and the second stage is assumed to be the stage in which COVID-19 is controlled in China, but spreads globally. Then, we will introduce baseline scenarios and the scenarios designed at these two stages, as shown in Figure 1.

In terms of baseline scenarios, as shown in Figure 1, baseline scenarios 1 and 2 assume that CTR do not import from China and meet demand for China’s products by increasing internal production (baseline scenario 1) or imports from ROW (baseline scenario 2). Baseline scenarios 3–5 assume that CTR do not import from China, while China does not import from CTR. Specifically, baseline scenario 3 assumes that CTR demand for China’s products and China’s demand for CTR products could be met by their own products; baseline scenario 4 assumes that CTR demand is met by ROW products and China’s demand is met by domestic products; and baseline scenario 5 assumes that CTR demand is met by their own products and China’s demand is met by ROW products.

At the first stage, China’s domestic outbreak may exacerbate trade protectionism. This means that the actual trade relations among regions at this stage may be closer to the extreme trade relations in the baseline scenarios. Therefore, this paper introduces the shock of the initial wave of the pandemic into the five baseline scenarios and defines them as scenarios 1–5, as shown in Figure 1. The trade relations in scenarios 1–5 correspond to those in baseline scenarios 1–5, respectively. At the second stage, the global spread of COVID-19 may further aggravate global trade protectionism. This paper introduces the shock of the second wave of the pandemic into baseline scenarios 3–5, and sets optimistic scenarios (scenarios 6–8) and pessimistic scenarios (scenarios 9–11) considering the uncertainty of the spread of the pandemic, as presented in Figure 1. The trade relations in scenarios 6–8 and scenarios 9–11 correspond to those in baseline scenarios 3–5, respectively.

Overall, we set 11 scenarios to simulate the shock of COVID-19 in the context of trade protectionism. Scenarios 1–5 at the first stage and scenarios 6–11 at the second stage are used to simulate the impacts of the pandemic in China and the global spread of COVID-19 in the context of trade protectionism, respectively.

### 4.3. Data for the COVID-19 Pandemic

To introduce the demand and supply shocks arising from COVID-19 into the optimization problem, we calculated the productivity loss rates and the decline rates of final demand in China under the initial and second waves of the pandemic.

(1) Data for the initial wave of the pandemic

Due to the lack of information on the productivity loss of China’s industries during the domestic outbreak of COVID-19, the productivity loss rates caused by the initial wave of the pandemic were estimated using the decline rates of value added of China’s industries in the first quarter of 2020. Following Zhou et al. [29], this paper converted the productivity loss rates in the first quarter of 2020 to those in the full year based on the annual shares of industrial value added in the first quarter of 2019.

Furthermore, due to the paucity of data for final demand change of China’s industries during the outbreak, the impacts of domestic outbreak on consumption in services and fixed asset investment were estimated by two indicators, i.e., the decline rates of total retail sales of consumer goods and fixed asset investment in the first quarter of 2020. First, using the annual shares of these two indicators in the first quarter of 2019, this paper converted the decline rates of consumption in services and fixed asset investment in the first quarter of 2020 to those in the full year. Then, based on these data for the full year, the decline rates of China’s final demand caused by the initial wave of the pandemic were estimated using the weight of the shares of final consumption expenditure by households and gross fixed capital formation in final demand. The basic data can be obtained from the National Bureau of Statistics of China.

(2) Data for the second wave of the pandemic

The Global Economic Prospects (GEP) released by the World Bank Group in January 2021 and the World Economic Outlook (WEO) released by the International Monetary Fund in October 2020 reported the GDP growth rates of countries in 2020. This paper used these data to estimate the range of the productivity loss rates in CTR and ROW caused by the second wave of the pandemic. First, based on the GDP growth rates of countries in 2020 reported by GEP and WEO, the weighted GDP decline rates of CTR and ROW were calculated using the GDP of countries in 2019 as weights. Then, due to the lack of information on the impacts of the global spread of COVID-19 on specific industries in regions, the GDP decline rates of CTR and ROW were appropriately adjusted to represent the productivity loss rates of various industries in these regions, according to the industry characteristics and the different effects of the initial wave of the pandemic on China’s industries. Finally, this paper sets optimistic scenarios and pessimistic scenarios under the second wave of the pandemic based on the range of the productivity loss rates of industries in CTR and ROW.

Moreover, due to the lack of data for final demand change of CTR and ROW during the spread of COVID-19, the growth rates of private consumption and fixed investment in emerging markets in 2020 reported by GEP were used to estimate the decline rates of final demand in CTR and ROW under the second wave of the pandemic. This paper used different data sources to calculate the decline rates of final demand in CTR and ROW because of the differences in countries covered by these two regions. Given the geographical location and the severity of outbreaks in countries covered by CTR, data for South Asia Region, Latin America and the Caribbean, Europe and Central Asia reported by GEP were used to estimate the impact of global spread of COVID-19 on CTR final demand. ROW consist of countries and regions in the world except for China and CTR. It should be noted that China, the main economy in East Asia and Pacific (EAP), has brought domestic outbreak under control while the pandemic continues to spread across the world, and China’s investment and consumption are recovering gradually. Therefore, to avoid a disruption in China’s demand recovery, this paper adopted data for emerging markets, except EAP, to estimate the impact of the global spread of COVID-19 on ROW demand.

## 5. Results and Discussion

**Figure 2:**
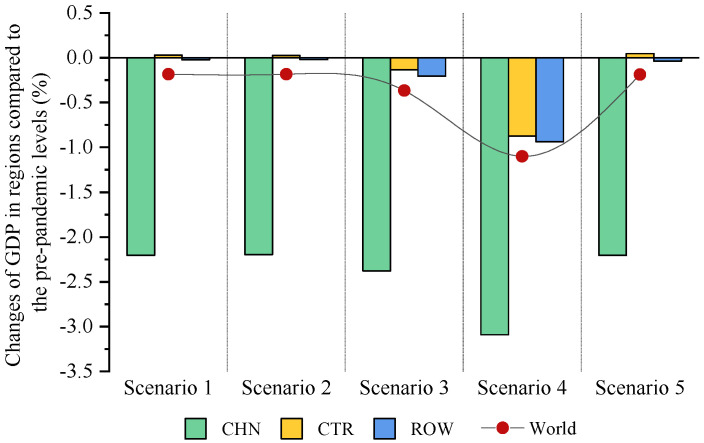
The impact of the COVID-19 outbreak in China on GDP in trade regions in the context of trade protectionism.

**Figure 3:**
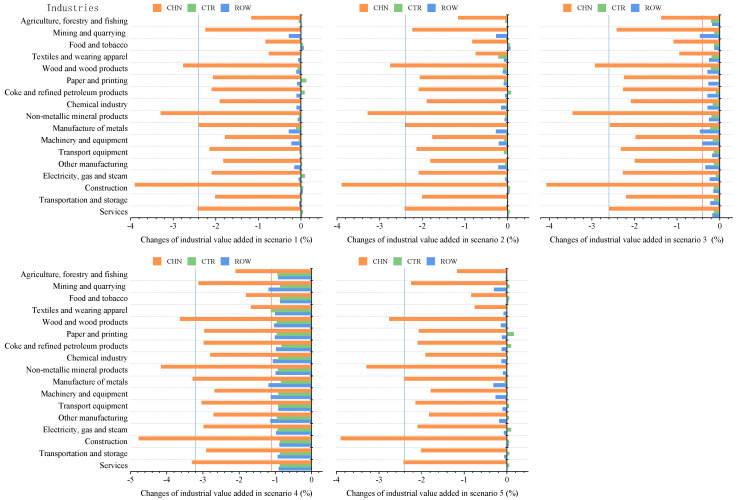
The impact of the COVID-19 outbreak in China on industrial value added in trade regions in the context of trade protectionism.

**Figure 4:**
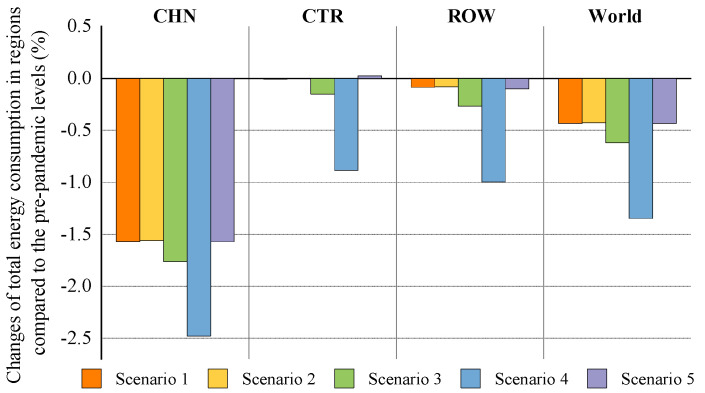
The impact of the COVID-19 outbreak in China on total energy consumption in trade regions in the context of trade protectionism.

**Figure 5:**
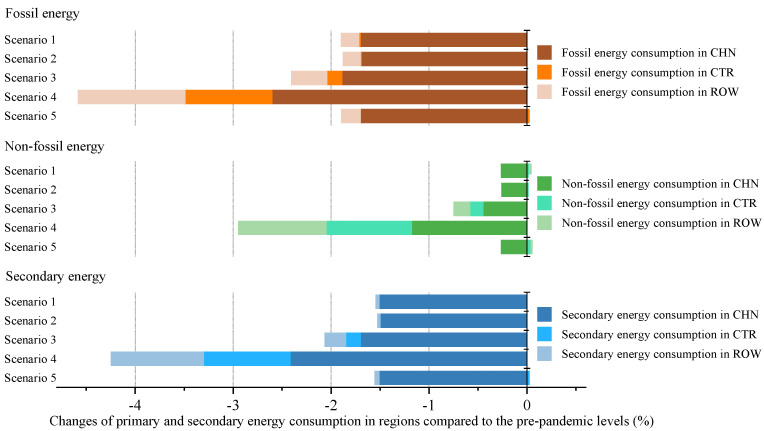
The impact of the COVID-19 outbreak in China on primary and secondary energy consumption in trade regions in the context of trade protectionism.

**Figure 6:**
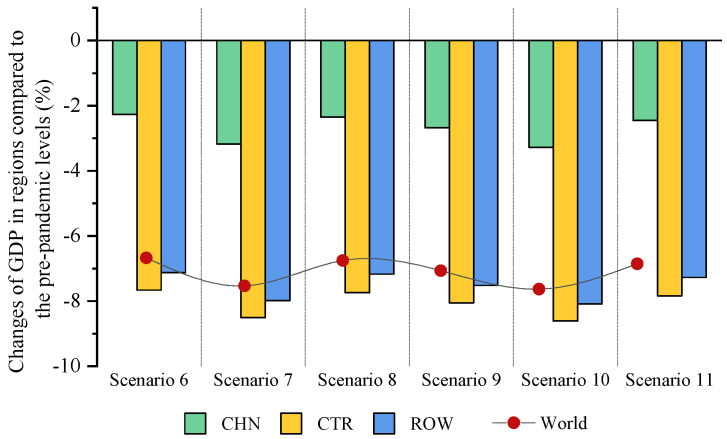
The impact of global pandemic spread on GDP in trade regions in the context of trade protectionism.

**Figure 7:**
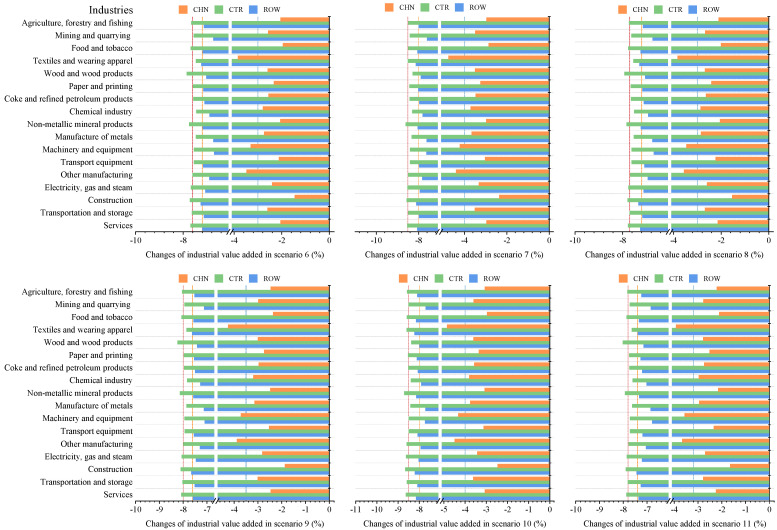
The impact of global pandemic spread on industrial value added in trade regions in the context of trade protectionism.

**Figure 8:**
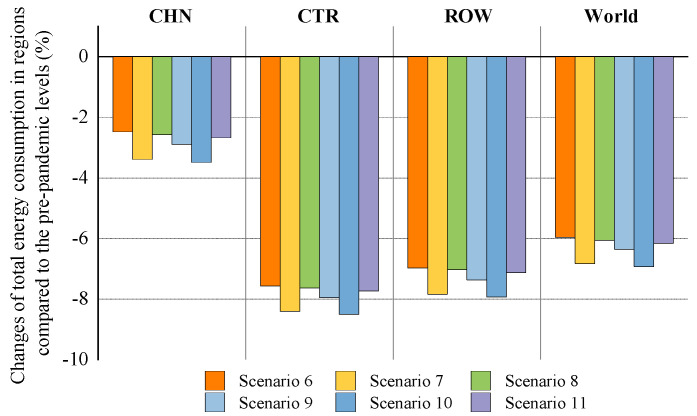
The impact of global pandemic spread on total energy consumption in trade regions in the context of trade protectionism.

**Figure 9:**
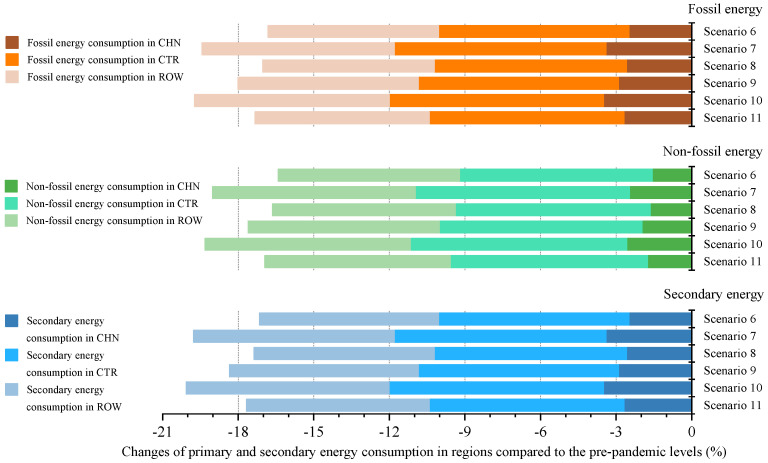
The impact of global pandemic spread on primary and secondary energy consumption in trade regions in the context of trade protectionism.

Based on the scenarios and stages defined in the previous section, this paper analyzes the impacts of COVID-19 on China’s economy and energy in the context of trade protectionism. The simulation results of scenarios 1–5 at the first stage are presented in Figures 2–5, showing the impacts of the COVID-19 outbreak in China in the context of trade protectionism. The results of scenarios 6–11 at the second stage are reported in Figures 6–9, which reflect the effects of global spread of COVID-19 in the same context.

### 5.1. The Impacts of the COVID-19 Outbreak in China on China’s Economy and Energy in the Context of Trade Protectionism

At the first stage, the COVID-19 outbreak in China not only had a direct impact on China’s economy and trade, but may also have prompted some countries to implement more trade restrictions. This paper sets scenarios 1–5 by introducing the shock of domestic outbreak into five baseline scenarios to assess the impacts of the outbreak on China’s economic development and energy consumption in the context of trade protectionism.

#### 5.1.1. The Impact of the COVID-19 Outbreak in China on GDP

Figure 2 presents the impact of the COVID-19 outbreak on China’s economy in the context of trade protectionism. Overall, the simulation result indicates that domestic outbreak will involve a 2.20–3.09% decline in China’s GDP relative to prepandemic levels. This finding is basically in line with those of previous studies such as Zhou et al. [29], who evaluated the macroeconomic effects of COVID-19 based on the CGE model and found that the pandemic would lead to a 1.43% drop in China’s GDP. In a similar study, Hu et al. [10] suggested that China’s GDP would fall by 1.27% under the optimistic scenario and by 2.07% under the pessimistic scenario during the pandemic. By contrast, the decline in GDP under the pandemic estimated in this paper is slightly higher than that estimated by Zhou et al. [29]. The main reason for this might be that the different settings of coefficients in the CGE model may lead to differences in the simulation results. For example, there are obvious differences in the estimates of impacts of the pandemic under the optimistic scenario and pessimistic scenario estimated by Hu et al. [10], and our estimates are much closer to their estimates in the pessimistic scenario. In short, the estimation results of these studies could, to some extent, support the credibility of the results of this study.

Furthermore, the COVID-19 outbreak in China may also affect the economic development in CTR and ROW in the context of trade protectionism. As shown in Figure 2, under scenarios 1, 2 and 5, economic growth in these two regions will be less affected by the outbreak, with GDP changing by less than 0.1%. In contrast, the outbreak in China will deliver a relatively large economic shock to CTR and ROW in scenarios 3 and 4. Under these scenarios, the channel of the outbreak’s economic impact ROW might be that the pandemic would cause severe economic losses in China, resulting in a contraction in its import demand for ROW. This would further negatively affect ROW economies. The negative shock of the outbreak to CTR may be due to the fact that ROW economic losses would indirectly affect CTR through trade between these two regions. Based on the above analysis, the possible explanations for the phenomenon that economic growth in CTR and ROW will be less affected by the outbreak under scenarios 1, 2 and 5 are as follows: Since ROW economies would be impacted by the pandemic through the trade between China and ROW, the magnitude of ROW economic losses depends largely on the magnitude of China’s economic losses. Under scenarios 1, 2 and 5, China’s economic losses were significantly less than those under scenarios 3 and 4, which might explain the smaller economic losses in ROW under these scenarios. Similarly, the impact of the pandemic on CTR is realized by affecting the trade between CTR and ROW. Under scenarios 1, 2 and 5, ROW will be less affected by the pandemic, meaning that trade between CTR and ROW is relatively stable under these scenarios. This is the reason why the outbreak in China would have a smaller impact on CTR under these three scenarios. Globally, the COVID-19 outbreak in China will lead to a decline in global GDP under scenarios 1–5. This suggests that in the context of the integration of the global economy, the outbreak in China would not only negatively affect China’s economy, but also generate adverse spillovers for other economies and the world.

#### 5.1.2. The Impact of the COVID-19 Outbreak in China on Industrial Value Added

As can be seen from Figure 3, in the context of trade protectionism, China’s industries will be negatively affected by this domestic outbreak, albeit in varying degrees. Of these industries, construction, non-metallic mineral products, wood and wood products, and services will suffer greater output losses, the decline rate in value added relative to them will average 1.29% higher than that of other industries. More concretely, in a scenario with the largest impact from COVID-19 on China’s economy (scenario 4), the value added of these four industries will fall by 4.77%, 4.15%, 3.63%, and 3.30%, respectively, relative to prepandemic levels; in a scenario with smaller impact from COVID-19 (scenario 2), the value added relative to them will decline by 3.89%, 3.28%, 2.75%, and 2.41%, respectively, compared to prepandemic levels. The reasons why these four industries would be greatly affected by the outbreak are as follows. First, as a labor-intensive industry, construction is vulnerable to production shutdowns, production delays, and labor shortages, together with the shortage of inputs and with transportation difficulties, making it the severely affected industry during the domestic outbreak. Second, non-metallic mineral products and wood and wood products, the upstream industries of construction and other industries, would be not only directly impacted by the pandemic, but also negatively affected by the output declines and investment weakness in their downstream industries. Third, the outbreak would sharply curb consumption of traditional services such as accommodation, food service, and tourism, but have little influence on emerging services such as financial services, and even drive the development of online services. The overall effect of the outbreak on services is negative as traditional services accounted for a larger proportion.

Figure 3 also shows the impacts of the COVID-19 outbreak in China on industry development in CTR and ROW. Since industry outputs in these two regions will be less affected by the COVID-19 outbreak under scenarios 1, 2, and 5, this paper analyses and discusses the impacts of the outbreak on industries based on the simulation results of scenarios 3 and 4. As can be seen from the graph above, mining and quarrying and manufacture of metals in ROW will be more negatively impacted by the outbreak. According to the WIOT 2014, ROW exports of these two industries accounted for 17.18% and 13.72% of the corresponding industry outputs, and their exports to China accounted for 44.84% and 42.42% of total exports of corresponding industries, respectively. It means that mining and quarrying and manufacture of metals are the main export industries in ROW, and China is the main export destination for these two industries. The COVID-19 outbreak in China would cause output losses in China’s industries, resulting in a contraction in its import demand for ROW products. This might be the main reason why these two industries in ROW would be greatly affected by the outbreak. In addition, the value added of CTR industries will decline to varying degrees in scenarios 3 and 4. This phenomenon indicates that even though these scenarios assume that trade between CTR and China stops, and CTR are not directly impacted by the outbreak in China, they would be indirectly affected through trade with ROW.

#### 5.1.3. The Impact of the COVID-19 Outbreak in China on Total Energy Consumption

Figure 4 shows that under scenarios 1–5, the domestic COVID-19 outbreak will cause a 1.56–2.48% drop in China’s total energy consumption in the context of trade protectionism, relative to prepandemic levels. This result may be explained by the fact that the COVID-19 shock could be transmitted from economic system to energy system through the interaction between economy and energy. After the pandemic hit, China adopted control measures such as shutdowns or delays of production and restrictions on transport. These measures delivered a significantly negative shock to economic activities, thus resulting in a substantial decline in domestic demand for energy products. The outbreak impact under scenario 4 would have the most severely adverse effect on China’s total energy consumption. This scenario’s simulation results, in Section 5.1.1, suggest that the outbreak would lead to large economic losses in China. This is the reason why there is a big drop in China’s total energy consumption under this scenario. Furthermore, the simulation results show that the decline in China’s GDP caused by domestic outbreak is slightly higher than that in its total energy consumption. The possible explanations for this are as follows: The production in some energy-intensive industries is related to the stability of people’s livelihood and the control and prevention of the pandemic. Furthermore, since energy demand in these industries is less affected by the outbreak, and human life has a rigid demand for energy products, the drop in energy consumption is less than that in GDP.

As illustrated in Figure 4, the changes of total energy consumption in CTR and ROW are less than 0.1% under scenarios 1, 2, and 5. Thus, this paper analyses the impact of the COVID-19 outbreak in China on their energy consumption based on the simulation results of scenarios 3 and 4. The results show that relative to prepandemic levels, the total energy consumption in CTR will fall by 0.15% and 0.89% in scenarios 3 and 4, respectively, and will decline, in ROW, by 0.27% and 1% under these two scenarios, respectively. There are two main reasons for the decline in ROW total energy consumption caused by China’s domestic outbreak. First, industry outputs in ROW would shrink due to the negative spillovers from the outbreak, which reduces their energy consumption as well. Second, the pandemic would cause a decline in China’s energy demand, with a contraction in its import demand for ROW energy products such as coal, oil, and natural gas. This would further steepen the drop in ROW energy consumption. Moreover, a major reason for the decline in CTR energy consumption is that industry outputs in CTR would be indirectly affected by the outbreak in China, thus resulting in a reduction in their energy demand. For the world, the simulation results suggest that the outbreak in China will involve a 0.43–1.35% decline in global energy consumption relative to prepandemic levels.

#### 5.1.4. The Impact of the COVID-19 Outbreak in China on Consumption of Energy Products

The simulation result of the domestic impact of the COVID-19 outbreak on China’s fossil energy and non-fossil energy consumption in the context of trade protectionism is presented in Figure 5. As can be seen from this figure, the COVID-19 outbreak will deliver a significantly negative shock to China’s fossil energy consumption, reducing it by 1.69–2.6% relative to prepandemic levels. In contrast, non-fossil energy consumption would be less impacted by the outbreak, with a 0.26–1.18% decline compared to prepandemic levels, a decline rate 1.44% lower than fossil energy consumption on average. There may be two reasons for this phenomenon. Firstly, the outbreak would affect China’s energy consumption mainly by hitting energy demand in energy-intensive industries such as the chemical industry, non-metallic mineral products, and manufacture of metals. Energy consumption of these energy-intensive industries is dominated by fossil fuels such as coal. Therefore, fossil energy consumption is more sensitive to the COVID-19 shock than non-fossil energy consumption. Secondly, China has provided a series of support policies for power generation from renewables to promote its development. These policies could offset to some extent the adverse effects from the pandemic on non-fossil energy demand. In addition, the finding that fossil energy consumption would be more affected by the outbreak is basically consistent with that of the Annual Report on China’s Energy Development 2019. The report shows that the pandemic would lead to a decline in China’s fossil energy consumption such as coal and oil, while non-fossil energy consumption would continue to grow, with a drop in growth rate. However, the result in this paper indicates that the outbreak would also hit China’s non-fossil energy consumption, which differs from the estimates in the report. Non-fossil energy is mainly used for power generation, heating, and biofuel production. It could be inferred that there are two reasons for the decline in non-fossil energy consumption: first, the outbreak would cause a fall in China’s electricity demand, thereby reducing the consumption of non-fossil fuels used for power generation; second, transportation would be hard hit by the pandemic, thus lowering the demand for biofuels.

Figure 5 also shows the impacts of the COVID-19 outbreak in China on fossil energy and non-fossil energy consumption in CTR and ROW in the context of trade protectionism. The simulation results show that under scenario 3, fossil energy and non-fossil energy consumption in CTR will decline by 0.15% and 0.14%, respectively, relative to prepandemic levels, and those in ROW will drop by 0.36% and 0.16%, respectively. Under scenario 4, those in CTR will fall by 0.89% and 0.87%, respectively, and those in ROW will decline by 1.09% and 0.90%, respectively. These results suggest that fossil energy consumption in both CTR and ROW would be slightly more impacted by the outbreak in China than non-fossil energy consumption, which is in agreement with the simulation result for China’s energy consumption. According to the WIOT, the shares of fossil energy in primary energy consumption in CTR and ROW were 92% and 89% in 2014, respectively. BP’s Statistical Review of World Energy 2020 reports that fossil energy still accounted for 84 % of global primary energy consumption in 2019. This could explain the phenomenon that fossil energy consumption in these regions would be greatly affected by the outbreak in China.

### 5.2. The Impacts of Global Spread of COVID-19 on China’s Economy and Energy in the Context of Trade Protectionism

At the second stage, COVID-19 continues to spread across the world, although in China the spread has been brought under control. This may aggravate global trade protectionism. With the deepening of China’s embedding in global value chains, the global spread of COVID-19 (simply global pandemic spread hereafter) and increased trade protectionism would have direct or indirect impacts on China’s economy and energy. By introducing the shock of the pandemic on CTR and ROW into baseline scenarios 3–5, this paper sets optimistic scenarios (scenarios 6–8) and pessimistic scenarios (scenarios 9–11) to evaluate the impacts of the global pandemic spread on China’s economic development and energy consumption in the context of trade protectionism.

#### 5.2.1. The Impact of Global Pandemic Spread on GDP

Figure 6 shows the impact of global pandemic spread on China’s economy in the context of trade protectionism. As can be seen from this figure, global pandemic spread will reduce China’s GDP by 2.27–3.18% under the optimistic scenarios (scenarios 6–8) and by 2.46–3.28% under the pessimistic scenarios (scenarios 9–11), compared with prepandemic levels. This means that although China has effectively brought domestic outbreak under control, the global pandemic spread would also generate adverse spillovers for China’s economy. The possible explanations for this phenomenon are as follows: Data from the National Bureau of Statistics of China show a high degree of China’s dependence on foreign trade, which was close to 32% in 2019. This implies that China is highly dependent on international markets and its growth is vulnerable to economic fluctuations in other economies. It could be inferred that global pandemic spread would cause cross-border spillovers to China through a negative impact on demand and supply in other economies. Concretely, first, the degree of export dependence in China is generally higher than that of import dependence, meaning that the negative spillover impacts of the pandemic on China come mainly from the demand side. Global pandemic spread would cause economic contractions in many countries, resulting in a decline in their demand for China’s products. This demand shock, together with disruptions to trade and transportation caused by pandemic-control measures, would deal a significant blow to China’s exports. Second, global pandemic spread would also negatively affect China’s economy through supply channels. In fact, some raw materials and crucial components needed by China’s manufacturing industry are highly dependent on imports. The pandemic spread would disrupt the production and supply of these products, thus leading to further output losses in the manufacturing industry. Moreover, it is found that the impact of global pandemic spread on China’s economy is slightly larger than that of the outbreak in China. COVID-19 rapidly struck the world in early 2020, the outbreaks in many countries were worse than that in China, which would lead to the economic recession in China’s major trading partners and in turn hit China significantly. This might be the main reason why global pandemic spread would deliver a larger economic shock to China than domestic outbreak.

Figure 6 indicates that global pandemic spread will reduce CTR GDP by 7.66–8.61% and ROW GDP by 7.13–8.08%, relative to prepandemic levels. By contrast, the declines in GDP of these two regions at this stage are roughly three times as steep as that of China’s GDP at the first stage. This implies that the adverse economic impacts of global pandemic spread on CTR and ROW are much larger than that of the domestic outbreak in China. This might be related to the severity of the outbreaks in different regions. In addition, the simulation results show that economic growth in CTR would be slightly more affected by global pandemic spread than that in ROW. The possible explanation for this is that CTR contain some countries with larger outbreaks, such as the United States, India, and Brazil, which were the three countries with the largest cumulative confirmed cases of COVID-19 as of 15 December 2020, according to real-time data of COVID-19. For the world, relative to prepandemic levels, global pandemic spread will involve a 6.66–7.62% decline in global GDP in the context of trade protectionism. This suggests that the pandemic would cause a deep global recession.

#### 5.2.2. The Impact of Global Pandemic Spread on Industrial Value Added

As shown in Figure 7, China’s industries will be negatively impacted by global pandemic spread in varying degrees in the context of trade protectionism. Of these industries, textiles and wearing apparel, machinery and equipment, and other manufacturing will suffer greater output losses, the decline rate in value added of them would be on average 1.23% higher than that of other industries. According to the analysis in Section 5.2.1, global pandemic spread would impact China’s economy mainly by hitting export demand. Therefore, China’s export-oriented industries such as textiles and wearing apparel, machinery and equipment, and other manufacturing will be significantly affected by the pandemic spread. Specifically, in the optimistic scenarios (scenarios 6–8), the value added of textiles and wearing apparel will fall by 3.82–4.75% relative to prepandemic levels, that of machinery and equipment by 3.30–4.21%, and that of other manufacturing by 3.48–4.39%; in the pessimistic scenarios (scenarios 9–11), the value added of these three industries will decline by 3.93–4.85%, 3.56–4.32%, and 3.66–4.49%, respectively, compared with prepandemic levels. The simulation results in Section 5.1.2 suggest that the COVID-19 outbreak in China will have the most severely negative impact on its construction, non-metallic mineral products, wood and wood products, and services. It is observed that industries more affected by global pandemic spread differ from those more affected by the domestic outbreak. This phenomenon might be explained as follows: At the first stage, domestic outbreak hit China’s economy through the supply and demand side. Thus, construction and manufacturing that are vulnerable to shutdowns and restricted labor supply, together with services that are vulnerable to consumption reduction, would suffer greater output losses caused by the outbreak. While at the second stage, the pandemic spread would generate negative spillovers for China’s economy mainly through demand-side channels, which delivers a significantly negative shock to its exports. This is the reason why export-oriented industries such as textiles and wearing apparel, and machinery and equipment would be more impacted by global pandemic spread.

Figure 7 shows that there are also differences in the impacts of global pandemic spread on various industries in CTR and ROW. In terms of CTR, construction, non-metallic mineral products, and services will be more negatively impacted by global pandemic spread, and the value added of them will fall by 7.71–8.65%, 7.75–8.70%, and 7.69–8.63%, respectively, relative to prepandemic levels. While for ROW, construction, textiles and wearing apparel, and services will be more affected by global pandemic spread, their value added will drop by 7.26–8.20%, 7.23–8.22%, and 7.19–8.15% compared to prepandemic levels, respectively. As can be seen, industries more affected by global pandemic spread in these regions are construction, manufacturing, and services, which are basically in line with industries in China more affected by domestic outbreak. This result may be explained by the fact that the basic characteristics of industries could determine to some extent the degree of the pandemic’s impacts on them. For example, construction, a labor-intensive industry, is vulnerable to shutdown and labor shortages, so construction in these three regions would be subject to severely adverse impacts; lockdowns and quarantines to slow the spread of the pandemic would dampen consumption of offline services such as accommodation and food service, making it the directly affected industry during the pandemic.

#### 5.2.3. The Impact of Global Pandemic Spread on Total Energy Consumption

The simulation result of the impact of global pandemic spread on China’s total energy consumption in the context of trade protectionism is presented in Figure 8. From this figure we can see that global pandemic spread will reduce total energy consumption in China by 2.48–3.39% under the optimistic scenarios (scenarios 6–8) and by 2.68–3.49% under the pessimistic scenarios (scenarios 9–11), relative to pre-pandemic levels. These results indicate that global pandemic spread would deliver a significantly negative shock to China’s energy consumption. There may be two reasons for this. In the first place, the negative spillover impacts of the pandemic spread will lead to a decline in export demand for China’s manufacturing industries, such as textiles and wearing apparel, and machinery and equipment. This would have a significant negative impact on manufacturing industries, resulting in a reduction in their energy demand. Secondly, according to the World Economic Survey (WES) database in 2014 and the China Statistical Yearbook in 2018, China exported energy products such as coke, kerosene, gasoline and diesel to some countries covered by ROW. ROW would suffer large economic losses from global pandemic spread. This would cause a contraction in ROW energy demand, thereby reducing their import demand for China’s energy products as well. To some degree, the above analysis could be supported by data from the CCS, which shows a decline in China’s exports of some energy products during the pandemic. For example, the cumulative amount of China’s exports of coke fell by 57% in 2020.

Figure 8 also presents the impact of global pandemic spread on energy consumption in CTR and ROW. Under scenarios 6–11, CTR total energy consumption will fall by 7.56–8.5% relative to pre-pandemic levels, and ROW by 6.98–7.93%. Global pandemic spread may affect ROW’ energy consumption through multiple channels. First, the pandemic spread would have a direct negative impact on outputs in ROW, resulting in a large drop in their energy consumption. Second, CTR would be also directly affected by the pandemic, with a decline in outputs, thereby leading to a severe contraction in their import demand for ROW energy products such as coal, oil, and natural gas. Third, China’s energy demand would shrink due to the negative spillovers of the pandemic spread, which reduces its demand for ROW energy products. Unlike ROW, there may be two channels for the pandemic’s impact on CTR energy consumption. The first is that the direct impact of the pandemic on CTR would cause a sharp decline in their energy demand, thus reducing the total energy consumption. On the other hand, energy demand contraction in ROW caused by global pandemic spread would reduce their import demand for CTR energy products such as oil and biofuels. For the world, relative to prepandemic levels, global pandemic spread will lead to a 5.96–6.93% reduction in global energy consumption in the context of trade protectionism.

#### 5.2.4. The Impact of Global Pandemic Spread on Consumption of Energy Products

As can be seen from Figure 9, in the context of trade protectionism, China’s fossil energy and non-fossil energy consumption will be affected by the global pandemic spread in varying degrees. The simulation result shows that relative to prepandemic levels, fossil energy consumption in China will decline by 2.49–3.4% under the optimistic scenarios (scenarios 6–8) and by 2.69–3.51% under the pessimistic scenarios (scenarios 9–11), while non-fossil energy consumption will drop by 1.56–2.47% under the optimistic scenarios and by 1.75–2.57% under the pessimistic scenarios. It implies that China’s fossil energy consumption is more sensitive to the shock of the pandemic spread; its decline rate would be 0.93% higher than non-fossil energy consumption on average. Three reasons might account for this. First, the energy consumption structure dominated by fossil energy could explain to some extent why fossil energy consumption would be more affected by the pandemic. Second, China’s support policies for renewables could help offset the negative impact of the pandemic on non-fossil energy demand. These two reasons are the same as the reasons why fossil energy consumption would be more impacted by domestic outbreak at the first stage. In addition, the third reason is that the pandemic spread would cause a contraction in ROW import demand for China’s energy products. The WES database in 2014 shows that China exported coke, natural gas and other fossil fuels to some countries covered by ROW. This means that weak external demand may lead to a decline in fossil energy consumption in China, but may not hit non-fossil energy consumption. In addition, this phenomenon is basically consistent with the characteristics of the impact of the outbreak in China on China’s primary energy consumption.

The simulation results show that global pandemic spread will affect fossil energy and non-fossil energy consumption in CTR and ROW. Overall, CTR fossil energy consumption will fall by 7.55–8.5% and ROW by 6.76–7.73%, while CTR non-fossil energy consumption will drop by 7.65–8.59% and ROW by 7.19–8.15%, compared with prepandemic levels. The World Energy Outlook 2020 released by the IEA suggests that global oil consumption was expected to decline by 8% and coal consumption by 7% in 2020. This could, to some degree, support the credibility of the declines, in these two regions, of fossil energy consumption estimated in this paper. Furthermore, the possible explanations for the phenomenon that non-fossil energy consumption in CTR and ROW would be more affected by the pandemic are as follows. On the one hand, according to the WES database in 2014, biomass accounted for more than 90% of renewable energy consumption in CTR and ROW. On the other hand, global pandemic spread may lead to a drop in biomass use for two reasons. First, the pandemic would cause interruptions or delivery delays of biomass power projects, resulting in a reduction in biomass use. Second, lower transport fuel demand caused by the pandemic, together with weaker competitiveness of biofuels due to a lowering of fossil fuel prices [53], would reduce the demand for transport biofuels, thereby leading to a decline in biomass use. Put differently, a high share of biomass in non-fossil energy consumption and a large impact of the pandemic on biomass use could explain the drop in non-fossil energy consumption in these two regions.

## 6. Conclusions and Policy Implications

This paper constructs an integrated economic and energy input-output model that includes the COVID-19 shock, and simulates and assesses the impacts of the pandemic on China’s economy and energy in the context of trade protectionism. The principal conclusions of this paper are as follows: Overall, the simulation results indicate that in the context of trade protectionism, domestic outbreak will lead to a 2.20–3.09% decline in China’s GDP, while global pandemic spread will cause a 2.27–3.28% drop in its GDP, compared to prepandemic levels. China’s industries will be negatively affected by two waves of the pandemic in varying degrees. Domestic outbreak would deliver a relatively large shock to construction, non-metallic mineral products, wood and wood products, and services, while global pandemic spread would have a larger negative impact on China’s textiles and wearing apparel, machinery and equipment, and other manufacturing.

Meanwhile, relative to prepandemic levels, the outbreak in China will reduce China’s total energy consumption by 1.56–2.48%, while global pandemic spread will cut that by 2.48–3.49%. For primary energy, these two waves of the pandemic would have a larger negative effect on China’s fossil energy consumption and a smaller effect on non-fossil energy consumption, with the effect on the former averaging 1.44% and 0.93% higher than the latter, respectively.

Based on these findings, the following policy implications can be obtained in this paper.

Firstly, China should pay more attention to problems in industry development exposed during the pandemic to promote the transformation and upgrading of traditional industries, and achieve high-quality development. The simulation results show that China’s industries with a low degree of digitalization, such as construction, traditional manufacturing, and services, will suffer greater output losses from the domestic COVID-19 outbreak. In contrast, industries with a high degree of digitalization such as e-commerce may be less affected by the outbreak. This reveals the problem of the lower degree of digitalization in some traditional industries. Hence, the outbreak should be considered as an opportunity to promote the transformation of traditional industries and accelerate the realization of high-quality development. One is to further increase the application of digital technology and intelligent construction technology in the construction industry, and propel the transformation of this industry in the direction of digital and intelligent aspects. The second is to comprehensively facilitate the deep integration of the internet, big data and artificial intelligence with the real economy, and promote the high-quality development of traditional manufacturing and services with automation, digitalization and intelligence.

Secondly, export-oriented industries in China should enhance their risk resistance and economic resilience to cope with the possible external demand shocks brought by the pandemic. This paper finds that since the global pandemic spread would impact China’s economy mainly by hitting export demand, export-oriented industries such as textiles and wearing apparel, machinery and equipment, and other manufacturing will suffer greater output losses. Therefore, China’s export-oriented industries should take active measures to cope with the external demand shock. On the one hand, export-oriented industries should increase R&D investment in high-end industries, improve industrial chain structure, and enhance China’s position in the global industrial chain. On the one hand, export-oriented industries could enhance their anti-risk capability by developing diversified export markets.

Thirdly, China should attach great importance to energy challenges posed by the pandemic, prevent risks relating to energy security, and ensure the stability and security of energy systems. The simulation results indicate that the pandemic would have a significant negative impact on fossil energy consumption, which might trigger a fall in global oil prices. As the second-largest oil consumer and the largest oil importer in the world, China would face challenges of energy security arising from the pandemic spread and the fall in global oil prices. In the first place, although lower oil prices would reduce China’s oil import costs and operating costs of the economy, they might lead to a drop in investment in the oil sector and weaken the competitiveness of renewable energy, which is detrimental to oil production, development of renewable energy and energy security. In the second place, the oil market outlook is subject to significant uncertainty as the duration of the pandemic remains unknown. This may even cause shortfalls in oil supply, threatening the security of energy supply in China. Against this background, China can take the following measures to ensure energy security. First, the country should further increase strategic oil reserves to enhance its ability to address emergency risks in global oil markets. Second, it is important to expand domestic oil and gas demand and develop strong policy support for oil and gas companies. These measures could alleviate the adverse impact of the pandemic on China’s oil sector and promote the secure and sustainable development of this sector. Third, vigorously developing renewables and reducing import dependence on fossil fuels could help to ensure energy security.

This study has the following limitations. Since the World Input-Output Table (WIOT) is only updated to 2014, this paper constructs the GMRIO model based on the WIOT 2014, and accordingly constructs the GEMRSU model based on the World Energy Statistics database in 2014. If the input-output data closer to the year of the COVID-19 outbreak can be used, the GMRIO model and the GEMRSU model could more precisely reflect the reality, which will more accurately describe the economic linkages and flow of energy products among various sectors in regions. This will better assess the impacts of the pandemic on China’s economy and energy in the context of trade protectionism. With the continuous updating of data, the data closer to the year of the outbreak of COVID-19 should be used in further research.

## Figures and Tables

**Figure A1:**
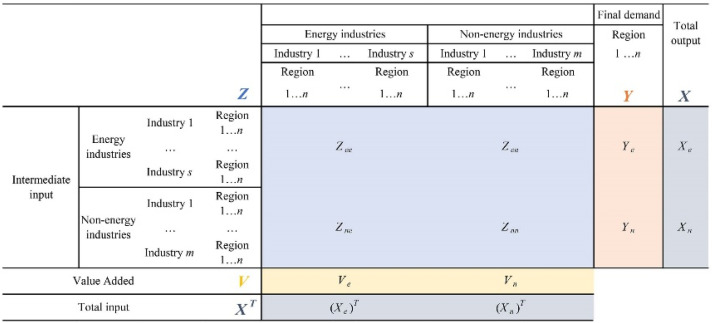
Structure diagram of the GMRIO table.

**Figure A2:**
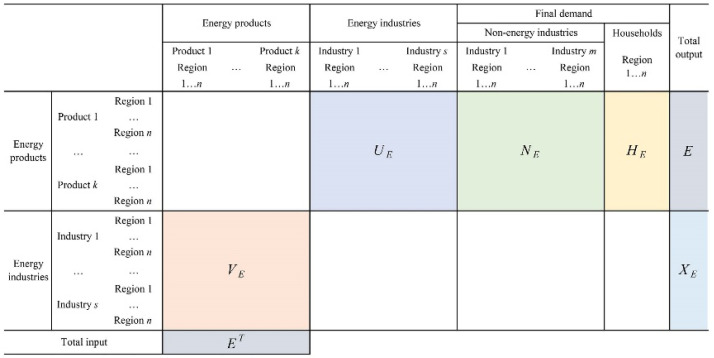
Structure diagram of the GEMRSU table.

## Data Availability

The data presented in this study are available on request from the corresponding author. The data are not publicly available due to some restrictions, and they are available on reasonable request.

## Appendix A. The GMRIO Model and the GEMRSU Model

### Appendix A.1. The GMRIO Model

The GMRIO model is constructed based on the World Input-Output Tables (WIOT), and the structure diagram of the GMRIO table is depicted in Figure A1.

In Figure A1, matrix *Z* is the multiregional interindustry flows matrix, matrix *V* denotes the multiregional value added matrix, matrix *Y* refers to the multiregional final demand matrix, and column vector *X* is the total outputs vector. Next, we adopt the Leontief–Kantorovich model to find an optimal resource allocation. This could provide the basis for simulating the impacts of external shocks on China’s economy and energy. The optimization problem could be described as follows: find an optimal resource allocation that could maximize final demand for a given level of primary resources, which can be expressed as

(A1) $$\begin{array}{l} \mathrm{Max}\;y=e^{\prime }\cdot Y\cdot e\\ s.t.\;(I-A)\cdot X\geq y\cdot s\cdot e\\ \;\;v\cdot X\leq V\cdot e\\ \;\;X\geq 0\\ \;\;y\geq 0 \end{array}$$

where, *y* represents the global final demand. Matrix *A* is the direct input coefficients matrix. Matrix *s* refers to the final demand structure matrix, *s* = *Y*⋅*y*^−1^. Matrix *v* denotes the input coefficients matrix of factors, *v* = *V*⋅($\hat{X}$)^−1^. *e* and *e*′ refer to the summation vectors of appropriate dimension. The total outputs *X* under the conditions of optimal resource allocation could be obtained by solving the optimization problem. Then, the changes in the value added matrix *V* can be calculated by the equation ∆*V* = ∆(*v*⋅$\hat{X}$) based on the changes in the total outputs *X*. Furthermore, this optimization problem only contains the primal resource allocation constraints. We will introduce other constraints according to the possible shock of COVID-19 to evaluate the pandemic’s impacts. A more detailed description of this is provided in Section 4.

### Appendix A.2. The GEMRSU Model

The physical GEMRSU table can be used to portray the energy conversion chain, and its structure diagram is shown in Figure A2.

In Figure A2, matrix *U_E_* refers to the use matrix of energy products, matrix *V_E_* denotes the make matrix of energy products, matrix *N_E_* and *H_E_* are the final demand matrices of energy products for non-energy industries and households, respectively. Column vector *X_E_* is the total outputs vector of energy industries. Column vector *E* refers to the total outputs vector of energy products. By defining the total requirements matrix of energy products in energy industries *L_E_*, the total outputs of energy products *E* can be written as:

(A2) $$E=L_{E}(N_{E}\cdot e+H_{E}\cdot e)$$

where *e* is the summation vector of appropriate dimension.

## Appendix B. Data for GMRIO Table and GEMRSU Table

GMRIO table is constructed using WIOT 2014, while GEMRSU table is constructed based on the World Energy Statistics (WES) database in 2014 released by the International Energy Agency (IEA). The concrete data processing and model construction are described in Wang and Wu [52].

## References

[1] Wang K., Tong J. (2020). The dynamic effect of trade protection barriers on export trade: Evidence from China’s HS-6 ex-port products to the US. Nankai Econ. Stud..

[2] Xu H., Chen Y.X., Ruan C.Y. (2019). Quantitative analysis method on international market segmentation based on fuzzy clustering model. Proceedings of the 2019 IEEE 4th International Conference on Cloud Computing and Big Data Analysis (ICCCBDA).

[3] Evenett S., Fiorini M., Fritz J., Hoekman B., Lukaszuk P., Rocha N., Ruta M., Santi F., Shingal A. (2021). Trade policy responses to the COVID-19 pandemic crisis: Evidence from a new data set. World Econ..

[4] Pinna A.M., Lodi L. (2021). Trade and global value chains at the time of COVID-19. Int. Spect..

[5] Ouyang Y., Li P. (2018). On the nexus of financial development, economic growth, and energy consumption in China: New perspective from a GMM panel VAR approach. Energy Econ..

[6] Shahbaz M., Zakaria M., Shahzad S.J.H., Mahalik M.K. (2018). The energy consumption and economic growth nexus in top ten energy-consuming countries: Fresh evidence from using the quantile-on-quantile approach. Energy Econ..

[7] Smith L.V., Tarui N., Yamagata T. (2021). Assessing the impact of COVID-19 on global fossil fuel consumption and CO_2_ emissions. Energy Econ..

[8] Norouzi N., Zarazua de Rubens G., Choupanpiesheh S., Enevoldsen P. (2020). When pandemics impact economies and climate change: Exploring the impacts of COVID-19 on oil and electricity demand in China. Energy Res. Soc. Sci.

[9] Wang Q., Su M. (2020). A preliminary assessment of the impact of COVID-19 on environment—A case study of China. Sci. Total Environ..

[10] Hu B., Fan Y., Zheng L. (2020). COVID-19, economic shock and government intervention. J. Quant. Tech. Econ..

[11] Choi S.Y. (2020). Industry volatility and economic uncertainty due to the COVID-19 pandemic: Evidence from wavelet coherence analysis. Financ. Res. Lett..

[12] Janus J. (2021). The COVID-19 shock and long-term interest rates in emerging market economies. Financ. Res. Lett..

[13] Jiang P., Fan Y.V., Klemes J.J. (2021). Impacts of COVID-19 on energy demand and consumption: Challenges, lessons and emerging opportunities. Appl. Energy.

[14] Brodeur A., Cook N., Wright T. (2021). On the effects of COVID-19 safer-at-home policies on social distancing, car crashes and pollution. J. Environ. Econ. Manag..

[15] Dang H.-A.H., Trinh T.-A. (2021). Does the COVID-19 lockdown improve global air quality? New cross-national evidence on its unintended consequences. J. Environ. Econ. Manag..

[16] Zhang L., Li H., Lee W.-J., Liao H. (2021). COVID-19 and energy: Influence mechanisms and research methodologies. Sustain. Prod. Consum..

[17] McKibbin W., Fernando R. (2021). The Global Macroeconomic Impacts of COVID-19: Seven Scenarios. CAMA Cent. Appl. Macroecon. Anal..

[18] Jawad M., Maroof Z., Naz M. (2021). Impact of pandemic COVID-19 on global economies (a seven-scenario analysis). MDE Manag. Decis. Econ..

[19] Madai Boukar A., Mbock O., Kilolo J.M.M. (2021). The impacts of the COVID-19 pandemic on employment in Cameroon: A general equilibrium analysis. Afr. Dev. Rev..

[20] Shan Y., Ou J., Wang D., Zeng Z., Zhang S., Guan D., Hubacek K. (2021). Impacts of COVID-19 and fiscal stimuli on global emissions and the Paris Agreement. Nat. Clim. Chang..

[21] Zhou L., Chen Z. (2020). Are CGE models reliable for disaster impact analyses?. Econ. Syst. Res..

[22] Sayan S., Alkan A. (2021). A novel approach for measurement and decomposition of the economywide costs of shutting down tourism and related service sectors against COVID-19. Tour. Econ..

[23] Bonet-Morón J., Ricciulli-Marín D., Pérez-Valbuena G.J., Galvis-Aponte L.A., Haddad E.A., Araújo I.F., Perobelli F.S. (2020). Regional economic impact of COVID-19 in Colombia: An input–output approach. Reg. Sci. Policy Pract..

[24] Huang R., Tian L. (2021). CO_2_ emissions inequality through the lens of developing countries. Appl. Energy.

[25] Munn I.A., Hussain A., Spurlock S., Henderson J.E. (2010). Economic impact of fishing, hunting, and wildlife-Associated recreation expenditures on the Southeast U.S. regional economy: An input–output analysis. Hum. Dimens. Wildl..

[26] Aruga K., Islam M.M., Jannat A. (2020). Effects of COVID-19 on Indian Energy Consumption. Sustainability.

[27] Shaikh I. (2021). Impact of COVID-19 pandemic on the energy markets. Econ. Chang. Restruct..

[28] Iqbal S., Bilal A.R., Nurunnabi M., Iqbal W., Alfakhri Y., Iqbal N. (2021). It is time to control the worst: Testing COVID-19 outbreak, energy consumption and CO_2_ emission. Environ. Sci. Pollut. Res. Int..

[29] Zhou M., Liu Y., Zhang J., Cui Q. (2020). COVID-19 and its macroeconomic countermeasures in China: Impact and effectiveness. J. Quant. Tech. Econ..

[30] Duan H., Bao Q., Tian K., Wang S., Yang C., Cai Z. (2021). The hit of the novel coronavirus outbreak to China’s economy. China Econ. Rev..

[31] Tan L., Wu X., Guo J., Santibanez-Gonzalez E.D.R. (2021). Assessing the Impacts of COVID-19 on the Industrial Sectors and Economy of China. Risk Anal..

[32] Wang K., Wang Y.W., Chang C.P. (2021). The impacts of COVID-19 pandemic on air pollution from energy consumption: Diverse evidence from China. Int. J. Green Energy.

[33] Wang Q., Li S., Jiang F. (2021). Uncovering the impact of the COVID-19 pandemic on energy consumption: New insight from difference between pandemic-free scenario and actual electricity consumption in China. J. Clean. Prod..

[34] Xu S., Liu Q., Lu X. (2021). Shock effect of COVID-19 infection on environmental quality and economic development in China: Causal linkages (Health Economic Evaluation). Environ. Dev. Sustain..

[35] Jia Z., Wen S., Lin B. (2021). The effects and reacts of COVID-19 pandemic and international oil price on energy, economy, and environment in China. Appl. Energy.

[36] Dube K., Nhamo G., Chikodzi D. (2021). COVID-19 cripples global restaurant and hospitality industry. Curr. Issues Tour..

[37] Wei X.L., Li L.J., Zhang F. (2021). The impact of the COVID-19 pandemic on socio-economic and sustainability. Environ. Sci. Pollut. Res..

[38] Bherwani H., Nair M., Musugu K., Gautam S., Gupta A., Kapley A., Kumar R. (2020). Valuation of air pollution externalities: Comparative assessment of economic damage and emission reduction under COVID-19 lockdown. Air Qual. Atmos. Health.

[39] Mofijur M., Fattah I.M.R., Alam M.A., Islam A., Ong H.C., Rahman S.M.A., Najafi G., Ahmed S.F., Uddin M.A., Mahlia T.M.I. (2021). Impact of COVID-19 on the social, economic, environmental and energy domains: Lessons learnt from a global pandemic. Sustain. Prod. Consum..

[40] Priya S.S., Cuce E., Sudhakar K. (2021). A perspective of COVID 19 impact on global economy, energy and environment. Int. J. Sustain. Eng..

[41] Salisu A.A., Adediran I.A., Gupta R. (2021). A note on the COVID-19 shock and real GDP in emerging economies. Emerg. Mark. Financ. Trade.

[42] Chudik A., Mohaddes K., Pesaran M.H., Raissi M., Rebucci A. (2021). A counterfactual economic analysis of COVID-19 using a threshold augmented multi-country model. J. Int. Money Financ..

[43] Li Z.Y., Farmanesh P., Kirikkaleli D., Itani R. (2021). A comparative analysis of COVID-19 and global financial crises: Evidence from US economy. Econ. Res.-Ekon. Istraz..

[44] Yousfi M., Ben Zaied Y., Ben Cheikh N., Ben Lahouel B., Bouzgarrou H. (2021). Effects of the COVID-19 pandemic on the US stock market and uncertainty: A comparative assessment between the first and second waves. Technol. Forecast. Soc. Chang..

[45] Su C.W., Dai K., Ullah S., Andlib Z. (2021). COVID-19 pandemic and unemployment dynamics in European economies. Econ. Res.-Ekon. Istraz..

[46] Goswami B., Mandal R., Nath H.K. (2021). COVID-19 pandemic and economic performances of the states in India. Econ. Anal. Policy.

[47] Ramakumar R., Kanitkar T. (2021). Impact of COVID-19 pandemic on the Indian economy: A critical analysis. Investig. Econ..

[48] Beck M.J., Hensher D.A. (2020). Insights into the impact of COVID-19 on household travel and activities in Australia—The early days of easing restrictions. Transp. Policy.

[49] Barichello R. (2020). The COVID-19 pandemic: Anticipating its effects on Canada’s agricultural trade. Can. J. Agric. Econ.-Rev. Can. D Agroecon..

[50] Ou S.Q., He X., Ji W.Q., Chen W., Sui L., Gan Y., Lu Z.F., Lin Z.H., Deng S.L., Przesmitzki S. (2020). Machine learning model to project the impact of COVID-19 on US motor gasoline demand. Nat. Energy.

[51] Rouleau J., Gosselin L. (2021). Impacts of the COVID-19 lockdown on energy consumption in a Canadian social housing building. Appl. Energy.

[52] Wang F., Wu M. How does trade policy uncertainty affect China’s economy and energy?. Struct. Chang. Econ. Dyn..

[53] IEA (2020). World Energy Outlook 2020.

